# Correction: Quantitative and Qualitative Perturbations of CD8^+^ MAITs in Healthy *Mycobacterium tuberculosis*–Infected Individuals

**DOI:** 10.4049/immunohorizons.2000064

**Published:** 2020-07-22

**Authors:** Mikhail Pomaznoy

Pomaznoy, M., R. Kuan, M. Lindvall, J. G. Burel, G. Seumois, P. Vijayanand, R. Taplitz, R. H. Gilman, M. Saito, D. M. Lewinsohn, A. Sette, B. Peters, and C. S. Lindestam Arlehamn. 2020.Quantitative and qualitative perturbations of CD8^+^ MAITs in healthy *Mycobacterium tuberculosis*–infected individuals. *ImmunoHorizons* 4: 292–307; DOI: https://doi.org/10.4049/immunohorizons.2000031.

The graphs in Fig. 5F were inadvertently duplicated from Fig. 6 in the article as originally published. The corrected Fig. 5 is shown below. The figure legend was correct as published and is shown below for reference. The figure has been corrected in the online article.

## Figures and Tables

**FIGURE 5.**
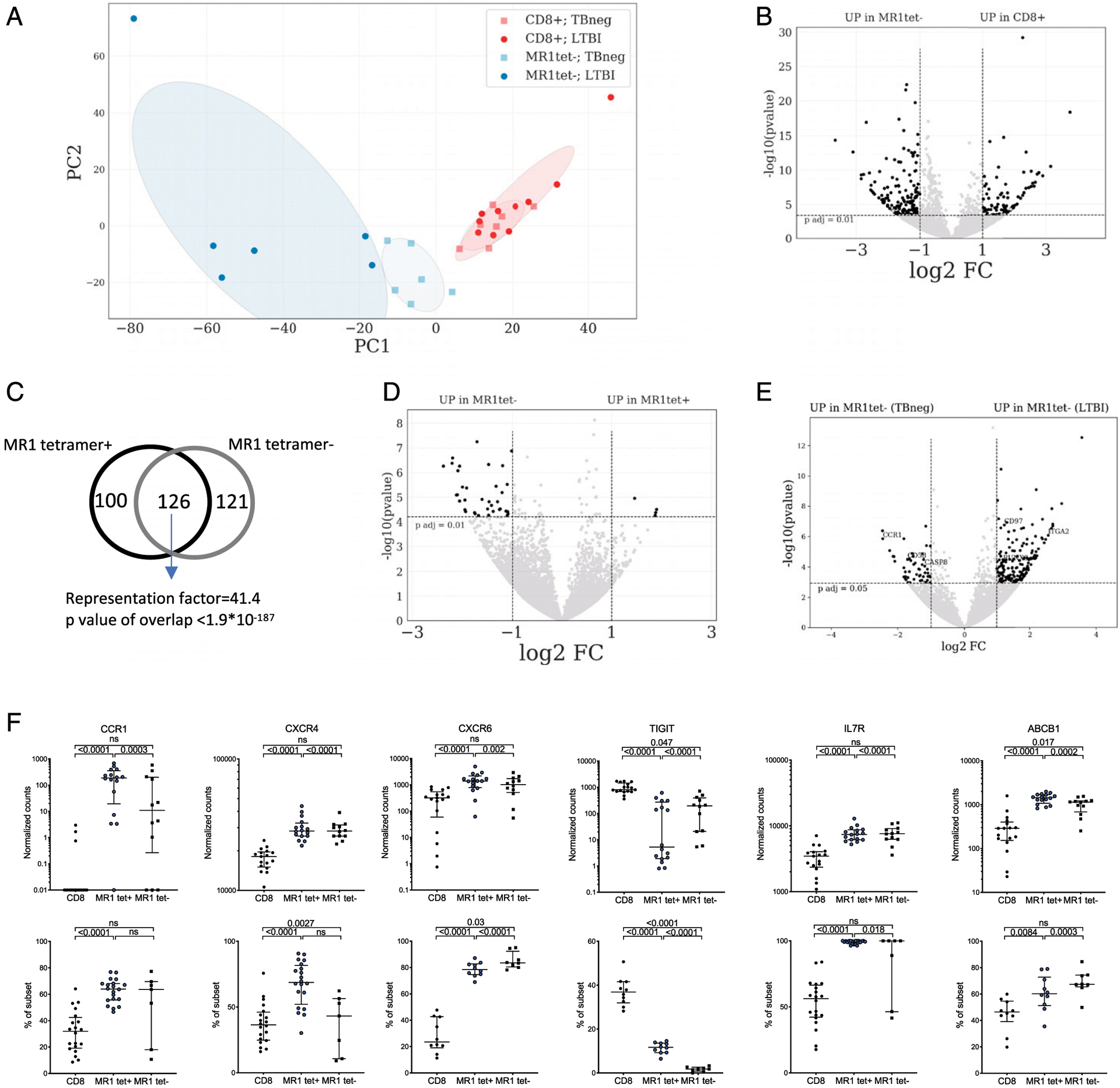
Gene expression profile and *M. tuberculosis*–specific signature of MR1tet^−^ MAITs compared with memory CD8 T cells and MR1tet^+^ MAITs. (**A**) PCA plot illustrating differences between memory CD8 T cells and MR1tet^−^ MAITs and between LTBI and TB neg individuals. (**B**, **D**, and **E**) Volcano plots obtained from the DEseq2 analysis showing log2 fold change versus −log10 *p* value. The differentially expressed genes are represented in black [adjusted *p* value <0.01 (B and E) and p < 0.05 (D), absolute log2 fold change >1 are indicated by dotted lines]. (B) MR1tet^−^ cells compared with memory CD8 T cells. (**C**) Venn-diagram showing overlap between the 226-gene signature identified in Fig. 4B and the signature in Fig. 5B, based on hyper-geometric distribution test (considering the 18,315 transcripts detected within memory CD8 T cells as the total number of genes). (D) MR1tet^−^ cells comparing individuals with LTBI versus TB neg. (E) Volcano plot comparing MR1tet^−^ cells with MR1tet^+^ cells. (**F**) CCR1, CXCR4, CXCR6, TIGIT, IL-7R, and ABCB1 expression at the mRNA (upper panels: gene expression values in counts normalized by sequencing depth calculated by the DEseq2 package) and protein (lower panels: protein expression as percent frequency of subset) levels in memory CD8 T cells and MR1tet^−^ MAITs. Gene expression data were derived from memory CD8 T cells from 17 individuals and MR1tet^−^ cells (*n* individuals = 12) using an Illumina sequencing platform. Protein expression data were derived from memory CD8 T cells from 20 individuals and MR1tet^−^ cells (*n* individuals = 7) using flow cytometry. Median ± interquartile range is shown. Two-tailed Mann–Whitney *U* test.

